# Sex and gender in hypertension guidelines

**DOI:** 10.1038/s41371-022-00793-8

**Published:** 2023-01-10

**Authors:** Fabian Meinert, Costas Thomopoulos, Reinhold Kreutz

**Affiliations:** 1 Charité – Universitätsmedizin Berlin, corporate member of Freie Universität Berlin and Humboldt-Universität zu Berlin, Institute of Clinical Pharmacology and Toxicology, Berlin, Germany; 2 Department of Cardiology, Helena Venizelou Hospital, Athens, Greece

**Keywords:** Hypertension, Health care

## Abstract

This paper reviews 11 current and previous international and some selected national hypertension guidelines regarding sex and gender-related differences. Those differences can be attributed to biological sex and to gender differences that are determined by socially constructed norms. All reviewed guidelines agree on a higher hypertension prevalence in men than in women. They also concur that evidence does not support different blood pressure thresholds and targets for treatment between men and women. Differences refer in addition to the differences in epidemiological aspects to differences in some morphometric diagnostic indices, e.g., left ventricular mass or the limits for daily alcohol intake. Concerning practical management, there are hardly any clear statements on different procedures that go beyond the consensus that blockers of the renin–angiotensin system should not be used in women of childbearing age wishing to become pregnant. Some further sex-specific aspects are related to differences in tolerability or drug-specific side effects of BP-lowering drugs. There is also a consensus about the need for blood pressure monitoring before and during the use of contraceptive pills. For management of pregnancy, several guidelines still recommend no active treatment in pregnant women without severe forms of hypertension, despite a wide consensus about the definition of hypertension in pregnancy. A disparity in treatment targets when treating severe and non-severe hypertension in pregnancy is also observed. Overall, sex-specific aspects are only very sparsely considered or documented in the evaluated guidelines highlighting an unmet need for future clinical research on this topic.

## Introduction

Hypertension is the leading modifiable risk factor associated with an increased cardiovascular disease and mortality rate [1]. Different guidelines for the management of hypertension have constantly acknowledged that the prevalence of hypertension is different between men and women across the lifespan [2, 3]. The varying prevalence of hypertension is related to biological (sex) and psychosocial (gender) factors and their interaction [4].

Biological sex is based upon different chromosome complements, generating differences in the molecular configuration of male and female cells. On a second level, random inactivation of one X chromosome in female cells may promote additional differences in gene expression between men and women [5]. Finally, in a third level, some X-linked genes escape inactivation in female individuals and may also have a higher degree of expression in women. In addition, the Y chromosomal SRY gene is fundamental for the development of testis which directs a surge of testosterone in male individuals. The testosterone excess, in turn, contributes to unique features of cellular gene expression in males that are associated with epigenetic differences at tissue and organ level between male and female individuals [5]. The combination of the above genetic, epigenetic, and developmental events determines sex differences in various biological measures and diseases, including blood pressure (BP) and hypertension.

Gender, according to the Global Health 50/50 definition, refers to socially constructed norms that impose and determine roles, relationships, and positional power for all people across their lifetime [6]. Gender interacts with sex, the biological and physical characteristics that define women, men, and those with intersex identities. Gender includes the belief that traits of masculinity or femininity may coexist and are expressed to a different extent. More than two-thirds of women and men report gender-related characteristics traditionally attributed to the opposite sex [7]. The distribution of gender-related characteristics within populations of men and women can influence health issues differently than biological sex [8]. Gender-related behaviors contribute to different risk exposure and preventive behavior in several diseases, including hypertension. However, in clinical studies of several diseases, including hypertension, the gender dimension has been largely ignored. Currently, efforts to discriminate the effect of sex and gender on disease burden are at least suboptimal or even misleading. Consequently, different guidelines for managing risk factors, such as hypertension, do not consider gender issues and report differences in disease diagnosis, treatment, or epidemiology between men and women.

We reviewed international and selected national guidelines for the management of hypertension representing all continents to underline between-guideline similarities and differences in epidemiology, diagnosis, and treatment of hypertension in men and women. We also aimed to explore how sex-related evidence evolved from earlier to recent hypertension international guidelines reports. Always under the guideline context, sex-specific conditions, such as hypertensive disorders in pregnancy, sexual dysfunction, menopause, or use of contraceptive pills, were also addressed.

## Methods

To address sex-related or sex-specific issues, we selected to review primarily the following international hypertension guidelines reported by: 1) the World Health Organization (WHO) in 2021 [9]; 2) the International Society of Hypertension (ISH) in 2020 [10]; 3) the American College of Cardiology (ACC)/American Heart Association (AHA) together with other scientific Societies from the United States of America (USA) in 2017 (ACC/AHA) [3]; 4) the European Society of Cardiology (ESC)/European Society of Hypertension (ESH) in 2018 (ESC/ESH) [2]; and 5) the Latin American Society of Hypertension in 2017 [11]. Furthermore, we reviewed also previous WHO and joint WHO/ISH guidelines [12–18], the Joint National Committee (JNC) I to JNC 7 guidelines from the USA [19–25], the ESH/ESC guidelines from 2003 to 2013 [26–28], and also recent national guidelines from the National Institute for Health and Care Excellence (NICE) in the United Kingdom (UK) in 2019 (https://www.nice.org.uk/guidance/ng136), from Australia in 2016 [29], from Korea in 2018 [30–32], from China in 2018 [33], and from Japan in 2019 [34]. In the guideline reports, we searched the following terms: (1) sex, (2) gender, (3) male, and (4) women to identify sections relevant to our investigational questions.

## Results

### 2021 WHO Guideline

Sex-related aspects and recommendations of previous WHO and joint WHO/ISH guidelines are presented in the Data Supplement [12–18]. In 2021, the WHO released a guideline paper for the pharmacological treatment of hypertension in adults [9].

#### Sex and gender aspects

Overall, it is recommended that pharmacological treatment of hypertension, including treatment initiation and targets for BP control, should not be different between men and women.

#### Pregnancy-related aspects

However, it was underlined that different drug treatment algorithms, including renin–angiotensin system blockers, are contraindicated during pregnancy and also in women who could become pregnant. Hypertension in pregnancy is generally diagnosed when BP is ≥140 mmHg and/or ≥90 mmHg on at least two occasions, at least six hours apart. Chronic hypertension is defined as a diagnosis of hypertension before 20 weeks of gestation, while gestational hypertension is defined as hypertension at 20 weeks or later. Preeclampsia and eclampsia are pregnancy-specific medical conditions requiring immediate and specific medical management. It was acknowledged that while BP treatment thresholds for hypertension in pregnancy continue to change, it is generally recommended for both chronic and gestational hypertension that pharmacologic treatment be initiated when the systolic BP is ≥160 mmHg and/or the diastolic BP is ≥105 mmHg. In women diagnosed with hypertension before pregnancy, the antihypertensive treatment may be continued unaltered. However, some medications may have to be changed to preferred medications, while medications contraindicated in pregnancy must be discontinued. The guideline report acknowledged that recommended on-treatment BP target in pregnancy also has been subject to debate and is changing. For instance, achieving lower BP target (diastolic BP of 85 mmHg vs. 100 mmHg) has decreased severe hypertension rates while not increasing maternal or fetal risk [35]. In the case of women with hypertension-mediated target organ damage, the initiation of antihypertensive treatment at a diastolic BP of ≥90 mmHg should be considered. Preferred medications are: methyldopa, beta blockers (particularly labetalol, but not atenolol), calcium channel blockers (particularly nifedipine and, as an alternative, verapamil), and direct-acting vasodilators (particularly hydralazine). There is evidence to suggest that among these agents, beta blockers and calcium channel blockers appear more effective than methyldopa in decreasing the development of severe hypertension later in pregnancy. Thiazide diuretics have been debated, particularly if the individual is already on a thiazide before pregnancy. In this situation, the thiazide diuretic may be continued.

### 2020 ISH Guideline

#### Sex and gender aspects

In 2020 the ISH issued a guideline report for the global management of hypertension [10]. According to this guideline, hypertensive men 55 or older and women 65 years or older are at higher risk of cardiovascular disease. Moreover, in a simplified classification algorithm of the overall cardiovascular risk, it is stated that risk categories will vary according to age and sex. Regarding alcohol consumption, recommended daily consumption of alcohol is limited to 20 g and 15 g of alcohol for men and women, respectively. Beta blockers should be used in younger women with or planning pregnancy. Fibromuscular dysplasia should always be suspected in women with onset of hypertension below 30 years.

#### Pregnancy-related aspects

Regarding the management of hypertension during pregnancy [10], identifiable hypertension-related conditions are pre-existing hypertension, gestational hypertension, pre-existing hypertension with superimposed gestational hypertension, preeclampsia, eclampsia, and HELLP (i.e., hemolysis, elevated liver enzymes, and low platelets) syndrome. 2020 ISH guidelines [10] endorsed the traditional definition of preeclampsia (i.e., hypertension and proteinuria). Optimally, ambulatory BP monitoring should be used to evaluate white coat hypertension, diabetes mellitus, and chronic kidney disease during pregnancy. Women at high risk of preeclampsia should be identified and treated with 75–162 mg daily aspirin. Drug treatment initiation is reserved for persistent BP of more than 150/95 mmHg for all pregnant women; however, a threshold of 140/90 mmHg may be used in women with hypertension-mediated organ damage. Methyldopa, slow-release nifedipine, nicardipine, and labetalol are preferred to treat mild hypertension. However, methyldopa should be avoided during puerperium, due to the risk of inducing postpartum depression. Hypertensive disorders in pregnancy are associated with an increased risk of hypertension and cardiovascular disease in later life.

### 2018 ESC/ESH Guideline

Sex-related aspects of previous ESC/ESH guidelines before 2018 are reported in Data Supplement [26–28]. The 2018 ESC/ESH guideline provides an extensive argumentation on sex-related or sex-specific issues for the management of hypertension [2].

#### Sex and gender aspects

The global age-standardized prevalence of hypertension is 24% and 20% in men and women, respectively. Male sex is associated with greater cardiovascular risk, which is incorporated into the Systematic Coronary Risk Evaluation (SCORE) system to estimate the 10-year fatal cardiovascular risk. However, the risk of total cardiovascular events compared to fatal events alone is approximately three times higher than the rate of fatal cardiovascular events in men and four times higher in women. The guideline further mentions different cut-off points for the diagnosis of left ventricular hypertrophy for men and women regarding electrocardiography (Cornell voltage) and echocardiography [2]. Sexual dysfunction may be triggered or aggravated by treatment with a thiazide or thiazide-like diuretic, conventional beta blockers, or centrally acting agents (e.g., clonidine).

In contrast, angiotensin converting enzyme inhibitors, angiotensin II receptor blockers, calcium channel blockers, or vasodilating beta blockers may have neutral or even beneficial effects. Moreover, very limited evidence suggests that antihypertensive medications are not associated with sexual dysfunction in middle-aged or older women. Spironolactone may not be well tolerated in men due to antiandrogenic side effects. However, it can also be associated with menstrual irregularities in women.

Hypertensive women are more likely to receive oral anticoagulation treatment, according to the CHA2DS2-VASc (Cardiac failure, Hypertension, Age ≥75 [Doubled], Diabetes, Stroke [Doubled] – Vascular disease, Age 65–74 and Sex category [Female]) algorithm. Alcohol consumption should be limited to 14 units per week in men and 8 units in women. Waist circumference should be <94 cm in men and <80 cm in women to prevent hypertension or reduce BP.

This guideline [2] also addressed the issue that combined estrogen–progesterone oral contraceptive pills can be associated with a small but significant increase in BP and the development of hypertension in about 5% of users. BP usually decreases promptly following cessation of these pills; consequently, BP should be monitored before and during oral contraceptive treatment. The 2018 ESC/ESH guideline [2] also acknowledges that older studies have demonstrated a relationship between oral contraceptive pills and venous thrombosis or thromboembolism and, to a lesser extent, myocardial infarction (especially with concomitant smoking history) and stroke. More recent studies with newer-generation oral contraceptive pills reported conflicting results. Thus, physicians prescribing oral contraceptives should consider the individual patient’s risks and benefits. Finally, hormone-replacement therapy and selective estrogen receptor modulators should not be used for primary or secondary prevention of cardiovascular disease, while there is no convincing evidence for a significant BP rise in menopausal women due to hormone replacement therapy.

#### Pregnancy-related aspects

A more expanded definition of different hypertensive disorders in pregnancy was attempted [2] compared to previous ESC/ESH guidelines [26–28], including (i) pre-existing hypertension, (ii) gestational hypertension, (iii) pre-existing hypertension plus superimposed gestational hypertension with proteinuria, (iv) preeclampsia, and (v) antenatally unclassifiable hypertension. Guidance on BP measurement in pregnancy at the office or at home with automated devices was reported, as well as the role of ambulatory blood pressure monitoring in pregnant women. To prevent preeclampsia, women at high or moderate risk of hypertensive disorders in pregnancy should receive 100–150 mg of oral aspirin daily from week 12 to week 36 of pregnancy. The 2018 ESC/ESH guidelines have recommended, despite paucity of evidence, initiating of drug treatment in all women with persistent elevation of BP ≥ 150/95 mmHg. Furthermore, pharmacological treatment is recommended in women with gestational hypertension, pre-existing hypertension (with the superimposition of gestational hypertension), or hypertension with subclinical hypertension-mediated target organ damage, when BP is >140/90 mmHg. A BP target of <140/90 was empirically suggested for pregnant women receiving antihypertensive therapy. In mild hypertension in pregnancy (140–159/90–109 mmHg), methyldopa, labetalol, and calcium channel blockers are drugs of choice; however, beta blockers may induce fetal bradycardia, and consequently, their type and dose should be carefully selected, with atenolol best avoided. Women with pre-existing hypertension may continue their current antihypertensive medication, while angiotensin converting enzyme inhibitors, angiotensin II receptor blockers, and direct renin inhibitors are, contraindicated due to adverse fetal and neonatal outcomes. Although BP-lowering is mandatory, the agents to use should be individualized for treating severe hypertension (≥160/110 mmHg). All antihypertensive drugs taken are excreted into breast milk. Most drugs are present at very low concentrations except for propranolol and nifedipine, with concentrations similar to those in maternal plasma. Finally, women who develop gestational hypertension or preeclampsia are at increased risk of hypertension, stroke, and ischemic heart disease in later life.

### 2017 ACC/AHA guideline

Sex-related aspects of previous JNC guidelines in the USA are reported in Data Supplement [19–25, 36, 37]. The 2017 ACC/AHA guidelines [3] also provided different sex-related aspects and recommendations.

#### Sex and gender aspects

Male sex was reported among cardiovascular risk factors in patients with hypertension. A gradient of progressively higher cardiovascular risk from normal BP to elevated BP and stage 1 or 2 hypertension is consistent across subgroups defined by sex. The estimated prevalence of hypertension is not different between the sexes by using the threshold of ≥140/90 mmHg (31% vs. 32%), while prevalence in men is somewhat higher compared to women (48% vs. 43%) when the threshold of ≥130/80 mmHg is applied, i.e. the cut-off levels for the definition of hypertension in the 2017 ACC/AHA guideline [3]. Prevalence of hypertension is lower in women than in men until about the fifth decade but is higher later in life. Another sex-related issue is that white-coat hypertension is higher in women than men. A higher incidence of angiotensin converting enzyme inhibitor-induced cough and edema with calcium channel blockers were observed in women. Diuretic use in women is more frequently associated with electrolytic disturbances, whereas gout is less frequent than in men. The prevalence estimates in men and women of hypertension awareness and treatment are higher in women. In contrast, hypertension control rates in those treated were not different between the sexes. Premature birth is associated with a 4-mmHg higher systolic BP and a 3-mmHg higher diastolic BP in adulthood, with somewhat larger effects in women [36]. Early-onset hypertension due to fibromuscular hyperplasia is more common in women. Limiting alcohol to ≤1 drink daily for women and ≤2 drinks for men was recommended. For women receiving oral contraceptives, the following recommendations were made: (1) use of low-dose (e.g., 20–30 mcg ethinyl estradiol) agents or a progestin-only form, or use of alternative forms of birth control where appropriate, and (2) use in women with uncontrolled hypertension should be avoided.

#### Pregnancy-related aspects

Focusing on special requirements for women in pregnancy, the 2017 ACC/AHA report [3] provided the following statements: (1) BP usually declines during the first trimester of pregnancy and then slowly rises, (2) the classification of hypertensive disorders in pregnancy included, (i) the newly pregnant mother with existing hypertension; (ii) incident hypertension; (iii) preeclampsia; and (iv) severe hypertension, (3) hypertension during pregnancy and preeclampsia are recognized as risk factors for future hypertension and cardiovascular disease, (4) the goal of antihypertensive treatment during pregnancy includes prevention of severe hypertension and prolonging gestation to allow the fetus more time to mature, (5) treatment of mild-to-moderate hypertension during pregnancy reduces the risk of developing severe hypertension, but has not be shown to prevent adverse pregnancy outcome including preeclampsia, (6) beta blockers (mainly labetalol) and calcium channel blockers (mainly nifedipine) appear superior to methyldopa in preventing preeclampsia, (7) it is recommended to screen all pregnant women for preeclampsia by measuring BP at every prenatal visit, (8) women with hypertension who become pregnant, or are planning to become pregnant, should be transitioned to methyldopa, nifedipine, and/or labetalol, (9) women with hypertension who become pregnant should not be treated with angiotensin converting enzyme inhibitors, angiotensin II receptor blockers, or direct renin inhibitors, (10) aspirin and magnesium should be used according to the American College of Obstetricians and Gynecologists task force report [38], while reference is made to another report detailing treatment of hypertensive emergencies during pregnancy and postpartum [39].

### 2017 Latin American Society of Hypertension Guideline

#### Sex and gender aspects

The 2017 report of the Latin American Society of Hypertension [11] states that the prevalence of metabolic syndrome in Latin America is higher in women than men (25.3% vs. 23.2%). Also, to define abdominal obesity, the cutoff values of waist circumference are 94 cm for men and 88 cm for women. In cases of renal artery stenosis in young individuals, fibromuscular dysplasia is more frequent in women.

#### Pregnancy-related aspects

Preferred drugs to treat mild hypertension in pregnancy are methyldopa, labetalol, nifedipine, or amlodipine. Drugs interfering with the renin-angiotensin system are forbidden and should not be prescribed to pregnant women or woman who are planning to get pregnant. Hypertension during pregnancy is defined as BP values higher than 140/90 mmHg when measured twice (same arm, interval of 15 min). Severe hypertension during pregnancy is defined as BP values higher than 160/110 mmHg. When systolic BP is at least 160 mmHg and/or diastolic BP at least 110 mmHg, BP reduction is mandatory. The Latin American guideline [11] recommends that the unique preeclampsia treatment is delivery. However, magnesium sulfate can be considered to prevent maternal convulsive events before and after delivery. Aspirin may be recommended in women at high risk of eclampsia before the onset or before the sixth week of pregnancy until delivery.

### Selected recent National Guidelines

Details of selected national guidelines including the 2019 NICE guideline (https://www.nice.org.uk/guidance/ng136, https://www.nice.org.uk/guidance/ng133), the 2016 guideline of the Australian Society of Hypertension [29, 40], the 2018 guideline of the Korean Society of Hypertension [30–32], the 2018 guideline of the Chinese Hypertension League [33], and the 2019 guideline of the Japanese Society of Hypertension [34] are presented in Data Supplement.

### Summary of findings

Sex-related aspects and recommendations in international and selective national hypertension guidelines are presented in Table 1. Gender issues are not reported in guidelines reports, while sexual dysfunction problems are largely underrepresented. In Table 2, we present aspects related to management of hypertensive disorders in pregnancy. Although there is consensus about the definition of hypertension in pregnancy, several guidelines still recommend not to treat non-severe hypertension. Furthermore, there is a disparity regarding treatment targets when treating severe or non-severe hypertension.

**Table 1:** Sex-related aspects and recommendations in hypertension guidelines.

Statements/recommendations	WHO 2021 [9]	ISH 2020 [10]	ESC/ESH 2018 [2]	ACC/AHA 2017 [3]	Latin American 2016 [11]	NICE 2019 [https://www.nice.org.uk/guideance/ng136]	Australian 2016 [29]	Japanese 2019 [34]	Chinese 2018 [33]	Korean 2018 [30–32]
Impact as modifier of baseline cardiovascular risk is lower in women	√	√	√	√	NR	NR	√	√	NR	√
Family history of premature CVD based on age-sex criteria	NR	No	√	No	NR	NR	√	√	√	√
Lifetime differences in hypertension prevalence between men and women	NR	NR	√	√	NR	√	√	√	√	√
Blood pressure trajectories in men and women during lifespan	NR	NR	√	NR	NR	NR	√	√	NR	√
Fibromuscular dysplasia more frequent in women	NR	√	√	√	√	NR	√	√	NR	√
Masked vs white-coat hypertension less frequent in women	NR	NR	√	√	NR	NR	NR	NR	NR	√
Drug-induced men sexual dysfunction issues	NR	NR	√	√	NR	NR	√	√	NR	√
Drug-induced women sexual dysfunction	NR	NR	√	NR	NR	NR	NR	NR	NR	NR
Sex-related side effects other than sexual dysfunction	NR	NR	NR	√	NR	NR	NR	√	NR	NR
Different upper limit of alcohol consumption in men and women	NR	√	√	√	NR	√	No	√	√	√
Beta-blockers as a treatment option in young women	NR	√	√	NR	NR	NR	NR	NR	NR	NR
Sex-related thresholds and targets of BP-lowering treatment	√	NR	√	√	NR	NR	NR	√	NR	√
Hormone replacement therapy	NR	NR	√	√	NR	NR	√	√	NR	√
Oral contraceptives	NR	NR	√	√	NR	NR	√	√	NR	√
Gender issues	NR	NR	NR	NR	NR	NR	NR	NR	NR	NR

*BP* blood pressure, *CVD* cardiovascular disease, *No* no sex-related statement or recommendation, *NR* not reported, √ existing statement or recommendation.

**Table 2:** Recommendations related to pregnancy in hypertension guidelines.

Aspects/recommendations	WHO 2021 [9]	ISH 2020 [10]	ESC/ESH 2018 [2]	ACC/AHA 2017 [3]^a^	Latin American 2016 [11]	NICE 2019 [https://www.nice.org.uk/guidance/ng133]^b^	Australian 2016 [29]^c^	Japanese 2019 [34]	Chinese 2018 [33]	Korean 2018 [30–32]
Definition of hypertension in pregnancy (mmHg)	≥140/90	≥140/90	≥140/90	≥140/90	≥140/90	≥140/90	≥140/90	≥140/90	NR	≥140/90
Classification of hypertensive disorders in pregnancy	√	√	√	√	NR	√	√	√	√	√
Aspirin to prevent preeclampsia in high-risk pregnancy	NR	Yes, 75–162 mg 12th week	Yes, 100–150 mg 12th week	Yes, 60–80 mg late 1st trimester	Yes, 100 mg, before 6th week	Yes, 75–150 mg 12th week	Yes, low dose	NR	Yes, low dose, 12th week	NR
Treatment of severe hypertension	√	√	√	√	√	√	√	√	√	√
Treatment of non-severe hypertension	If comorbidities	√	√	No	No	√	Individualize	No	√	√
On-treatment BP target of severe hypertension (mmHg)	NR	NR	NR	140–150/90–99	NR	130–150/80–100	NR	<160/110	NR	<150/100 Not <80
BP threshold to initiate drug treatment in non-severe hypertension (mmHg)	≥160/105 Diastolic >90 (if comorbidities)	>150/95	>150/95 or >140/90 if comorbidities	<160/110 only if comorbidities	≥160/110	≥140/90	≥140/90	NR	≥150/100	>150/95
On-treatment BP target of mild hypertension (mmHg)	NR	NR	<140/90	NR	NR	<135/85 Not <110/70	NR	NR	<150/100 Not <130/80	<150/100 Not <80
Preferred agent(s) to treat severe hypertension	NR	iv labetalol iv esmolol iv urapidiliv nicardipine	iv labetalol po nifedipine	iv hydralazine po nifedipine	NR	iv labetalol po nifedipine iv hydralazine	iv labetalol po nifedipine iv hydralazine	iv nicardipine iv nitroglycerin iv hydralazine	NR	iv labetalol
Preferred agents to treat non-severe hypertension	po labetalol po nifedipine po verapamil po methyldopa po hydralazine	po labetalol po nifedipine po nicardipine po methyldopa	po labetalol po calcium channel blockers	po labetalol	po labetalol po methyldopa po nifedipine po amlodipine	po labetalol po methyldopa po nifedipine po enalapril^b^	po labetalol po methyldopa po nifedipine po oxprenolol	po labetalol po methyldopa po nifedipine^d^ po hydralazine^e^	po labetalol po methyldopa po nifedipine	po labetalol po methyldopa po nifedipine

*BP* blood pressure, *NR* not reported.

^a^2013 American College of Obstetricians and Gynecologists’ guideline.

^b^To treat hypertension during puerperium.

^c^2014 Society of Obstetric Medicine of Australian and New Zealand (SOMANZ) guideline.

^d^After the 20th week.

^e^As an alternative agent in gestational hypertension.

## Discussion

### General aspects

From the epidemiological point of view, all available guidelines agree that hypertension prevalence is higher in men. The difference is mediated by the higher prevalence in men until 50 years, while women have the same or even higher prevalence after this age [2, 3]. The 1978 WHO guidelines [12] already indicated a common age threshold to indicate a positive family history of cardiovascular disease in first-degree relatives of hypertensive patients for females and males, that is, 65 years. However, all subsequent reports endorsed a differential age threshold of 55 years for men and 65 years for women. Hypertensive men aged 55 years or older and women aged 65 years or older are at higher risk, with no additional risk factors. However, the age-related thresholds stratified by sex were lower by 10 years in the Korean hypertension guidelines [30–32].

International and different national guidelines for the management of hypertension agree that men are more likely to present with masked rather than white-coat hypertension. There is consensus between guidelines that current evidence does not support differential BP thresholds for treatment and targets between the sexes. Regarding lifestyle measures, it is recommended that waist circumference be reduced according to sex-specific criteria that are different between Asian and non-Asian populations. In most cases, the recommended upper threshold to define unhealthy alcohol consumption stratified by sex differs between guidelines. Finally, although several guidelines indicated that fibromuscular dysplasia is highly more prevalent in women than men, any differences in the prevalence of other secondary causes of hypertension including primary hyperaldosteronism were not approached.

Guidelines agree that the outcome benefits from BP-lowering treatment are not different for the same extent of BP reduction. The sex-specific analysis of the Systolic Blood Pressure Intervention Trial (SPRINT) has shown that the primary composite endpoint was reduced by 16% in women and by 27% in men with no interaction between treatment and sex. The lack of statistical interaction indeed suggests that the overall SPRINT data are not grossly different between men and women [41], in line with the relevant guidelines’ statement in the field. However, the preferred use of BP-lowering agents may differ between men and women. For example, renin-angiotensin system blockers are not recommended in women of childbearing age. In addition, because sexual dysfunction, especially in men, might be related to or aggravated by some drug classes (e.g., use of diuretics or beta blockers) [2, 42], guidelines suggest a careful clinical assessment before prescribing drugs in sexually active men [2]. However, sexual dysfunction in hypertension and the response to BP-lowering pharmacological treatment may be relevant also in women [43].

A previous guideline report [25] raised concern that specific drug side effects are more frequently observed in women (e.g., hypokalemia with diuretics, dry cough with angiotensin converting enzyme inhibitors, peripheral edema with calcium channel blockers). Unfortunately, definitive evidence of sex differences in adherence to antihypertensive therapy cannot be drawn. Our little knowledge about factors affecting adherence, particularly sex effect among the elderly, urgently requires high-quality studies investigating these issues [44].

Regarding women-specific issues, at least in current guidelines [2, 3], there is consensus that hormone-replacement therapy should not be used to prevent cardiovascular disease. Because contraceptive pills can be associated with a small increase in BP and a higher risk of thromboembolic events, assessment of baseline cardiovascular risk and BP monitoring before pill initiation is advised. There is a paucity of the association between polycystic ovary syndrome and hypertension in different guidelines [45].

#### Pregnancy-related aspects

Although most of the hypertension guidelines have a limited or expanded section of recommendations on hypertensive disorders during pregnancy, several areas of disparity have been reviewed elsewhere [45]. Briefly, there is no consensus and/or lack of information on (1) the definition of preeclampsia components that remain by and large unspecified, (2) the manifestations of fetal complications in hypertension disorders in pregnancy, (3) the predictive value of biomarkers on adverse pregnancy outcomes, and (4) the definition of severe preeclampsia. Although recent guidelines [2, 3, 10] recommend the use of low-dose oral aspirin to prevent preeclampsia in high-risk pregnant women, there is a lack of consensus about (1) how preeclampsia risk can be identified, (2) which dose of aspirin to use, and (3) when treatment with aspirin should be started or stopped during pregnancy. Previous and recent guidelines largely agree that drugs affecting the renin-angiotensin system have been associated with serious fetal toxicity, including renal and cardiac abnormalities and death. During pregnancy, hypertension must be diagnosed, treated (when appropriate), and followed up diligently because of the adverse consequences to the woman and fetus. In some guidelines pregnancy related hypertension is also mentioned to be associated with a higher risk of cardiovascular events in later life [2, 3, 9, 30–32]. Although there is a general agreement that drug treatment of severe hypertension during pregnancy is mandatory, the type and route of administration of different drugs and the on-treatment BP target vary across guidelines. Dissimilarities are also detected in the treatment of non-severe hypertension concerning the choice of the first-line agent, the BP threshold to initiate BP-lowering treatment, and the BP target to which BP should be reduced [46].

#### Gender-related aspects

The role of gender in hypertension guidelines is by and large ignored. The term “gender” was used for the first time in the 1988 JNC IV [22] and 1993 WHO guidelines [16] to replace biological sex and not to define the interrelated psychosocial factors hidden behind biological sex. However, gender mostly disappeared from current guidelines because of lacking evidence in the field. Hypertension guidelines report, however, social and psychological epidemiological data, e.g., lower vs. higher-income individuals or countries, rural vs. non-rural areas, and lower vs. higher educational patient levels. However, this type of evidence was not stratified by the biological sex to generate “gender attitudes.” Beyond the guideline paucity about gender-related epidemiological aspects of hypertension, there is lacking clinical guidance on transgender medicine issues for the effects on BP levels or cardiovascular events of drug treatments to change sex to an acquired gender. This is in specific relevant to testosterone therapy with lacking consent on the effects on BP levels [47].

## Conclusion

Overall, the ESC/ESH guideline provides the most information about sex-specific aspects, followed by the ACC/AHA guideline, but in general those aspects are only very sparsely considered or documented in all evaluated hypertension guidelines highlighting thus an unmet need for future clinical research on this topic. This may include a thorough review of all clinical aspects of hypertension and particularly research on differences in the time-course of hypertension and development of sex-specific phenotypes of hypertension and organ damage. Future individual-patient data meta-analyses may also shed more light on sex-related BP-lowering outcome effects, including differential BP targets. Furthermore, studies with newer-generation oral contraceptive pills are desirable to define whether the new pill formulations are associated with BP increase and cardiovascular events. In addition, future trials in pregnancy are necessary to establish the optimal BP thresholds and targets to pursue following pharmacological treatment to reduce the rate of adverse pregnancy outcomes. In this regard, forthcoming guidelines should redefine the different phenotypes of hypertensive disorders in pregnancy and endorse a broader definition of preeclampsia. They may also draft recommendations on the association between hypertensive disorders in pregnancy and assisted reproductive technologies [48].

## Supplementary information

Data Supplement
