# Supplementary material for: Sex and gender in hypertension guidelines
Source: J Hum Hypertens. 2023 Jan 10;37(8):654–61. doi: 10.1038/s41371-022-00793-8 (PMC10403347; doi:10.1038/s41371-022-00793-8)
Supplement: Supplementary file 1 — Data Supplement [file 41371_2022_793_MOESM1_ESM.docx]

**DATA SUPPLEMENT**

**Sex and Gender in hypertension guidelines**

**Previous WHO and WHO/ISH guidelines**

*1978*

*Sex and gender aspects*

In 1978, the WHO reported that although a rise in BP with age occurs both in men and women, the BP increase appears more evident in women after age 50 [13]. Also, the higher the BP, systolic or diastolic, the greater the cardiovascular morbidity and mortality in both sexes. A common age threshold of 65 years was considered for both men and women to indicate a positive family history of cardiovascular disease and, therefore, a higher risk. Oral contraceptives were considered drugs to increase BP virtually in all women, especially when containing at least 50 μg of estrogens. Thus, oral contraceptives were included in the pharmacological agents causing hypertension list.

*Pregnancy-related aspects*

1978 WHO report classified hypertensive disorders during pregnancy among causes of secondary hypertension where an identifiable cause is underlying BP elevation. It has been recommended that the phase IV of Korotkoff be used in women during pregnancy to identify diastolic BP. The diagnosis of hypertensive disorders in pregnancy did not mandate the presence of hypertension. It was based on the presence of at least two components: hypertension, proteinuria, or peripheral edema. It has also been emphasized that during pregnancy, a diastolic BP of 85 mmHg or higher, irrespective of systolic BP, and a diastolic BP rise of at least 15 mmHg compared to pre-pregnancy levels should be considered abnormal. In such cases, physicians should first advise bed rest, salt restriction, and regular clinical monitoring in pregnant women with hypertensive disorders. Subsequently, judicious use of antihypertensive agents should be offered whenever the conservative measures do not suffice.

*1983/1986/1989*

The first report of the WHO/ISH guideline for managing mild hypertension was published in 1983 [14]. Although no sex-related issues were discussed in the first report.

*Sex and gender aspects*

In the first WHO/ISH update released in 1986 [15], a recommendation for hypertensive women to use alternative methods of contraception in place of oral contraceptives was made. In 1989, a second WHO/ISH update [16] reported that women have a lower risk of cardiovascular disease than men, and the evidence of benefit from BP-lowering treatment is less strong in women.

*Pregnancy-related aspects*

In addition, angiotensin converting enzyme inhibitors may increase the risk of early fetal death and should be avoided in women considering childbearing.

*1993/1996*

*Sex and gender aspects*

In 1993, a third WHO/ISH update [17] underlined that premenopausal women are at a 50% lower risk of cardiovascular disease than men of the same age. The absolute benefit of treatment is less conspicuous in this group of women than in men unless other risk factors are present. Furthermore, in men, hypertension appears to be frequently underdiagnosed and undertreated. Male sex was recognized as a cardiovascular risk factor favoring antihypertensive treatment because men are at higher risk of cardiovascular disease than women. In postmenopausal hypertensive women, there is no contraindication using hormone replacement therapy. However, BP should be monitored more frequently, as it was not yet clear whether hypertensive responses may occur in some women.

In 1996, a technical report from the WHO [18] was released on hypertension control. Male than female sex was considered a more important risk determinant, especially for future coronary events in patients with hypertension. This report also emphasizes BP trajectories in men and women. It was supported that no significant BP difference between the sexes exists in early life. Beginning at adolescence, however, men tend to display a higher average BP level than women. The between-sex BP difference is more evident in young and middle-aged than in older adults. Late in life, the difference in BP narrows, and the pattern may even be reversed. While this change late in life is partly accounted for by the higher premature death rates of middle-aged men with high BP, postmenopausal changes in women also may be contributory. It has been stated that long-term studies and large clinical trials of antihypertensive treatment have included both men and women but have not demonstrated sex differences in BP response and outcomes. However, the same report acknowledged that because the rate of cardiovascular events in middle-aged women is much lower than in men, these trials had limited power to distinguish the degree of benefit from treatment in men and women. Oral contraceptives, especially those with an estrogen concentration of at least 50 μg, may increase systolic to a higher and diastolic BP to a lower extent. However, some women may experience a marked BP elevation. Stopping oral contraceptives may reduce BP to non-hypertensive levels in most women, but this may take six or more months. Recommendations for oral replacement therapy were not different compared to previous WHO-initiated reports but have further observed that 1) hormone replacement treatment may prevent osteoporosis, 2) estrogen-alone may have beneficial effects on coronary events, and 3) combined treatment of estrogen and progesterone in women with an intact uterus may be protective against uterine malignancy.

*Pregnancy-related aspects*

The WHO-guideline legacy to report pregnancy-mediated BP disorders among secondary causes of hypertension was extended to the 1996 WHO report.However, at variance with the 1978 WHO hypertension guideline [13], some changes in managing hypertensive disorders in pregnancy appeared in 1996 WHO report [18]. It was suggested that the prevalence of a hypertensive pregnancy is less than 5% in the general population of pregnant women. Moreover, the definition of a hypertensive pregnancy is based on an average diastolic BP value of 90 mmHg or more by considering two or more BP measurements four or more hours apart or one BP measurement of at least 100 mmHg. Measurements of BP in pregnancy require a mercury sphygmomanometer. Women should lay on their sides at an angle of 15-30 degrees to the horizontal. The phase IV of Korotkoff should be used as previously suggested in the 1978 WHO guideline [13]. A classification of hypertensive disorders in pregnancy was attempted, including four different phenotypes: 1) preeclampsia/eclampsia, 2) chronic hypertension of whatever cause, 3) chronic hypertension with superimposed preeclampsia/eclampsia, 4) transient or late hypertension. The definition of preeclampsia changed compared to the 1978 WHO guideline [13], with hypertension being the *sine qua non*-criterion for the disease diagnosis together with proteinuria. Consequently, peripheral edema was not considered among the criteria. Low-dose aspirin use was not recommended to prevent preeclampsia because of inadequate evidence. Instead, magnesium sulfate should be used to prevent eclampsia. Hydralazine should intravenously be administered in severe diastolic hypertension associated with preeclampsia targeting diastolic BP levels between 90 and 100 mmHg. Salt restriction should not be advised during pregnancy, while methyldopa and beta blockers were considered the first-line agents at diastolic BP levels of 95 mmHg or higher.

*1999*

*Sex and gender aspects*

In 1999 the WHO/ISH released a new guideline [19] in which sexual dysfunction should be noted in the comprehensive assessment of clinical history, while diuretics may represent a possible contraindication in the drug selection procedure for sexually active males. It has also been reported that, at most ages, the risk of cardiovascular disease is greater in men than women. However, this difference declines with increasing age and seems greater for coronary heart disease than for stroke. An additional epidemiological remark was that the male sex is associated with poorer BP control than the female sex. Male sex was also considered among the risk factors increasing the absolute individual risk of future cardiovascular events at any given level of BP. Moreover, the phenotypes of women below 65 years or men below 55 years with grade 1 hypertension and no other risk factors should be considered in the low cardiovascular risk group. The argumentation related to hormone replacement therapy was not different compared to the previously WHO-released guidelines [13,18]. Regarding lifestyle measures, hypertensive patients who drink alcohol should be advised to limit their consumption to no more than 20-30 g of ethanol per day for men and no more than 10-20 g per day for women.

*Pregnancy-related aspects*

In the 1999 WHO/ISH guideline [19], hypertension in pregnancy was usually defined either by an absolute level of BP (e.g., 140/90 mmHg or greater) or by a rise in BP from preconception or first trimester levels (e.g., systolic BP rise ≥25 mmHg and/or diastolic BP rise ≥15 mmHg). At variance with the 1996 WHO report [18], the phenotype of transient/late hypertension in pregnancy was omitted. It is clearly stated that aspirin is not effective in preventing preeclampsia, according to the limited available evidence. Finally, a multidisciplinary team should ideally manage hypertensive disorders in pregnancy. Maternal and fetal monitoring is essential for detecting signs of advancing pre-eclampsia or impending fetal demise and, thereby, the need for delivery, which remains the definitive management for pre-eclampsia. No different recommendations for selecting antihypertensive agents in pregnancy were offered in 1999 WHO/ISH report [19] compared to the 1996 WHO report [18].

**Previous ESC/ESH guidelines**

*2003*

*Sex and gender aspects*

In 2003, the ESC and ESH produced the first joint guideline paper for the management of hypertension [27]. It has been proposed that the age-dependent stratified by sex cardiovascular risk is higher for men of 55 years or more and women 65 years or older. Although the risk for cardiovascular events, particularly coronary heart disease, differs greatly between men and women, based on individual patient data analysis, the risk ratios between the treated and control groups did not differ between men and women, regardless of the outcome. Consequently, there are no significant interactions between treatment effects and gender, so the proportional reduction of the cardiovascular risk appears to be similar in women and men.

Regarding the assessment of cardiac hypertrophy, although the relation between the left ventricular mass index and cardiovascular risk is continuous, a differential threshold of 125 g/m^2^ for men, and 110 g/m^2^ for women, is most widely used for conservative estimates of left ventricular hypertrophy. At the kidney level, it was recommended that the estimated creatinine clearance calculated on the basis of gender be measured in all hypertensive patients; however, the presence of mild renal insufficiency has been defined as serum creatinine values equal to or above 133 mmol/l (1.5 mg/dl) in men and 124 mmol/l (1.4 mg/dl) in women. It has also been proposed that a differential threshold for abdominal obesity should be applied to men and women. Hypertensive patients who drink alcohol should be advised to limit their consumption to 20-30 g ethanol/day for men and 10-20 g ethanol/day for women.

*Pregnancy-related aspects*

Regarding pregnancy issues, the 2003 ESC/ESH guideline [27] described the BP changes during pregnancy. It was stated that BP falls physiologically in the second trimester, reaching a mean of 15 mmHg lower than levels before pregnancy. It returns to or may exceed the pre-pregnancy levels in the third trimester. These fluctuations in BP are observed in all pregnant women (i.e., normal BP levels and women with chronic or gestational hypertension). Regarding the definition of hypertension during pregnancy, the 2003 ESC/ESH guideline [27] discarded the relative elevations of BP during the second trimester from a baseline reading in the first trimester or to pre-pregnancy levels, preferring absolute BP values (systolic BP > 140 mmHg or diastolic BP > 90 mmHg). The guideline suggested confirming high BP readings on two occasions and recommended that both phase IV and V Korotkoff sounds be recorded. Phase IV should be used for initiating clinical investigation and management. The proposed classification of hypertensive disorders was the following: 1) pre-existing hypertension, 2) gestational hypertension, 3) pre-existing hypertension plus superimposed gestational hypertension with proteinuria, and 4) antenatally unclassifiable hypertension. A normal diet without salt restriction was advised, while low-dose aspirin may, however, be used prophylactically in women with a history of early-onset (<28 weeks) preeclampsia. Regarding pharmacological treatment in pregnant women with chronic hypertension, the 2003 ESC/ESH guideline acknowledged that the benefit remains largely undetermined. While there is a consensus that drug treatment of severe hypertension in pregnancy is required and beneficial, treatment of less severe hypertension is controversial. In addition, the guideline stated that intravenous hydralazine should no longer be considered the drug of choice because it is associated with more perinatal adverse effects than other drugs. As such, they recommended that in cases of mild hypertension, antihypertensive treatment should be offered at the threshold of >140/90 mmHg to women with a higher risk profile at variance with a more conservative threshold of >150/95 mmHg to be reserved to lower risk women. The drugs of choice for women with non-severe hypertension are methyldopa, labetalol, calcium channel blockers, and beta blockers. It has also been recommended that the administration of calcium channel blockers should be avoided in women receiving magnesium sulfate because of a potential hypotension risk. In the case of breast-feeding, the guideline [27] support that 1) it does not increase BP in the nursing mother, 2) all antihypertensive agents taken by the nursing mother are excreted into breast milk, and 3) most of the antihypertensive drugs are present at very low concentrations, except for propranolol and nifedipine, concentrations of which are similar in breast milk to those in maternal plasma.

*2007*

*Sex and gender aspects*

The following are the new or changing sex-related items in the 2007 ESC/ESH [28] compared to the 2003 ESC/ESH guideline [27]. First, albuminuria should be related to urinary creatinine excretion, applying sex-specific criteria. Second, even low estrogen oral contraceptives are associated with an increased risk of hypertension, stroke, and myocardial infarction. A progestogen-only pill is a contraceptive option for women with high BP, but the influence on cardiovascular outcomes has been insufficiently investigated. Third, hormone replacement therapy is associated with decreased incidence of bone fractures and colon cancer. However, it is also accompanied by an increased risk of coronary events, stroke, thromboembolism, breast cancer, gallbladder disease, and dementia. This therapy should not be recommended for cardioprotection in postmenopausal women. Finally, the 2007 ESC/ESH guideline [28] report that adrenal adenomas are most common in women than men, while isolated office hypertension is more common in women than men.

*Pregnancy-related aspects*

Fourth, in high-risk pregnant women with hypertension, or particularly those with diabetic or renal damage, for both diagnostic and treatment purposes, it may be useful to perform ambulatory BP monitoring. Fifth, during pregnancy, the Korotkoff phase V is recommended for the measurement of diastolic BP, with phase IV being indicated if Korotkoff sounds persist at cuff pressures approaching 0 mmHg. Angiotensin converting enzyme inhibitors and angiotensin II receptor blockers should be avoided in fertile women or immediately withdrawn in case of pregnancy. Women with a history of hypertensive disorders during pregnancy seem to be at increased risk for cardiovascular disease in later life.

*2013*

*Sex and gender aspects*

In 2013, another ESC/ESH hypertension guideline report was issued [29]. The new or changing aspects of sex-related issues compared to previous ESC/ESH guideline versions [27,28] were the following: 1) male gender is a risk factor for masked hypertension, 2) the evidence for the effects of hypertension and antihypertensive therapy on female sexual dysfunction is quite limited, 3) fibromuscular dysplasia is more frequent in women than men, 4) total alcohol consumption should not exceed 140 g per week for men and 80 g per week for women, 6) the incidence of gynecomastia with spironolactone is dose-related whereas the exact incidence of menstrual disturbances in premenopausal women with spironolactone is unknown.

*Pregnancy-related aspects*

Women at high risk of preeclampsia (i.e., women hypertension in a previous pregnancy, chronic kidney disease, autoimmune disease such as systemic lupus erythematosus, or antiphospholipid syndrome, type 1 or 2 diabetes or chronic hypertension) or with more than one moderate risk factor for preeclampsia (first pregnancy, age >40 years, pregnancy interval of >10 years, BMI >35 kg/m^2^ at first visit, family history of preeclampsia and multiple pregnancy), may be advised to take 75 mg of aspirin daily from 12 weeks until the birth of the baby, provided that they are at low risk of gastrointestinal hemorrhage.

**JNC guideline**

*1977/1980/1984*

*Sex and gender aspects*

In 1977, the first release of the JNC guideline on detecting, evaluating, and treating high BP [20] proposed that antihypertensive treatment should be reserved for patients with diastolic BP of 105 mmHg or higher. However, male sex or other risk factors (i.e., elevated systolic BP, presence of target organ damage, family history of cardiovascular disease, smoking, elevated cholesterol levels, and diabetes mellitus) may influence the decision to start antihypertensive treatment at lower diastolic BP levels (i.e., between 90 and 105 mmHg). In the second version [21] of the JNC guideline (i.e., JNC II), no sex-related differences for hypertension management were provided. However, it has been suggested that sexual dysfunction may occur with BP-lowering by central or peripheral adrenergic inhibiting agents. The JNC III guideline [22] expanded the list of BP-lowering treatment drugs associated with sexual dysfunction beyond adrenergic system blockers. The new list members were thiazide diuretics, amiloride, and spironolactone. Although in the JNC III [22], the recommendation to treat diastolic hypertension was restricted to patients with diastolic BP of 95 mmHg or higher, male sex was included among the cardiovascular risk factors to consider for treatment initiation to mildly increased diastolic BP levels (i.e., 90-94 mmHg).

*Pregnancy-related aspects*

The JNC III guideline [22] also suggested that pregnancy-induced and pre-existing hypertension should be treated with methyldopa or beta blockers to control BP during pregnancy and improve fetal survival.

*1988*

*Sex and gender aspects*

The JNC IV guideline [23] continued to underline that different drug categories may impair sexual function, especially by inducing impotence in men. Also emphasized the role of the male sex as a risk modifier associated with an increased cardiovascular risk to mandate antihypertensive drug treatment initiation to diastolic BP levels from 90 to 94 mmHg.

*Pregnancy-related aspects*

The pregnancy-related recommendations of the JNC IV guideline were: 1) women with pre-existing hypertension should not stop BP-lowering treatment, 2) renin- angiotensin-system blockers are contraindicated during pregnancy, and 3) the preferred antihypertensive drugs during pregnancy are methyldopa and hydralazine. Finally, although it has been reported that calcium channel blockers proved effective in controlling severe hypertension in late pregnancy, beta blockers were not considered among the agents to use during pregnancy at variance with the JNC III guideline [22].

*1993*

*Sex and gender aspects*

The JNC V guideline [24] acknowledge that sexual dysfunction in men has been reported as a side effect of using all antihypertensive agents of that time. At the same time, limited data were available for the effect of antihypertensive drugs on sexual function in women. A sex-oriented epidemiological view was offered in the JNC V guideline [24] by stating that in young adults and early middle-aged individuals, high BP prevalence is greater for men than for women, and after that, the reverse is true. An increased waist-to-hip ratio above 0.85 in women and 0.95 in men has been correlated with hypertension. The JNC V guideline [24], for the first time, clearly stated that, according to the available evidence from large clinical trials, there is insufficient data to support a different approach to managing hypertension in women. Additional women-related topics were discussed in the JNC V guideline [24]. First, the use of contraceptives is related to BP increase, usually within the normal range. Their use for 5 years or more has been associated with at least a two times higher incidence of hypertension than women not taking contraceptives. Second, women aged 35 years and older who smoke should be strongly counseled to stop smoking. If they continue to smoke, they should be discouraged from using oral contraceptives because most cardiovascular deaths attributable to oral contraceptive use have been in such women. Third, in women who develop hypertension during treatment with contraceptives, it is advisable on an individual basis to stop the use of the pill because the BP will normalize in most cases within a few months. For postmenopausal women, the JNC V report [24] recommended that the use of estrogen replacement treatment can be used in women with hypertension and may have a beneficial effect on BP and overall cardiovascular risk. However, because a few women may experience a rise in BP attributable to estrogen therapy, it is recommended that all women treated with hormonal replacement have their BP monitored more frequently after such therapy is instituted.

*Pregnancy-related aspects*

Compared to all previous JNC guideline reports [20-23], the JNC V [24] provides detailed recommendations for the management of hypertensive disorders during pregnancy. JNC V endorses four diagnostic categories to differentiate and define hypertensive disorders (i.e., chronic hypertension; preeclampsia/eclampsia; chronic hypertension with superimposed preeclampsia; transient hypertension). According to the JNC V report [24], the arbitrary criteria for diagnosing hypertension in pregnancy are a systolic BP increase of 30 mmHg or greater and a diastolic BP increase (Korotkoff phase V) of 15 mmHg or greater compared with the average values before 20 weeks gestation. However, a reading of 140/90 mm Hg or above is abnormal when previous BP is unknown. For pregnant women who were not taking antihypertensive therapy in the early stages of pregnancy, increased rest or stopping work may be helpful when diastolic BP is between 90 and 100 mmHg. Moderate sodium restriction should be considered if it was useful before pregnancy. Antihypertensive drug therapy is reserved for pregnant women with diastolic BP greater than 100 mmHg. Aggressive antihypertensive therapy is discouraged because of the concern for maintaining adequate uteroplacental blood flow. Regarding agents to be used, methyldopa was considered the first-line agent. At the same time, beta blockers may be equally effective and safe during the second part of the pregnancy because their use at earlier stages may be associated with fetal growth retardation. However, hydralazine, calcium channel blockers, and a-blockers are useful additions or alternatives. Therapy for preeclampsia consists of hospitalization with bed rest, control of BP, seizure prophylaxis when signs of impending eclampsia are present, and timely delivery. At that time, because of limited data, the use of labetalol was not recommended. At the same time, aspirin at an ultra-low dose (i.e., 60 mg/day) may be administered in women at high risk of hypertensive complications in pregnancy.

*1997*

*Sex and gender aspects*

The JNC VI report [25] stated the following sex-related opinions: 1) postmenopausal women were considered at higher cardiovascular risk together with men of any age compared to women of age before menopause, 2) family history of cardiovascular disease is considered significant at a different age for men or women (55 or 65 years, respectively), 3) BP-lowering treatment was not associated with differential outcome effects between men and women at least in the elderly, 4) sex does not affect responsiveness to various BP-lowering agents, 5) definition of hypertension in children is different between boys and girls stratified by age and height, 6) angiotensin converting enzyme inhibitors and angiotensin II receptor blockers should not be used in sexually active girls, and 7) alcohol consumption should be 50% lower for women than men for hypertension prevention and management.

*Pregnancy-related aspects*

Regarding the treatment of hypertensive disorders in pregnancy, no different recommendations were provided in the JNC VI report [25] compared to the JNC V [24].

*2003*

*Sex and gender aspects*

The novel sex-related aspects introduced in the JNC 7 report [26] compared to previous JNC reports [20-25] were the following: 1) sexual dysfunction in men treated with thiazides is more pronounced with the higher doses of the drug, 2) a better description of lifetime BP trajectories in men and women was provided, 3) although the evidence from BP-lowering trials supports that the relative risk reduction in the overall cardiovascular risk was not different between men and women, the absolute risk reduction of coronary heart disease was higher in men than women, whereas it was not different for stroke events, 4) clinicians should be willing to discuss sexual dysfunction problems and offer counseling to improve the patient’s quality of life, 5) heart failure with preserved ejection fraction is more likely in hypertensive women than men, 6) chronic kidney disease defined in terms of estimated glomerular filtration rate corresponds to higher serum creatinine value in men than women, 7) endorsement of the Third Report of The National Cholesterol Education Program (NCEP) Expert Panel on Detection, Evaluation, and Treatment of High Blood Cholesterol in Adults (Adult Treatment Panel III) sex-related criteria [37] specifically for high-density lipoprotein cholesterol and waist circumference to define metabolic syndrome, 8) women are more likely than men to know that they have hypertension, to have it treated, and to have it controlled, 9) the important role of menopause on BP changes has been underlined, 10) women with current hormonal replacement treatment are more likely diagnosed with hypertension than women with previous or no exposure to the same treatment, 11) although diuretics may be potentially beneficial to older women because of the prevention of hip fractures, side effects of different drugs may be more pronounced in women than men: e.g., diuretics for hypokalemia; cough for angiotensin converting enzyme inhibitors; peripheral edema for calcium channel blocker, and 12) in patients, usually women, with fibromuscular dysplasia, results of percutaneous transluminal renal angioplasty have been excellent and comparable to surgical revascularization.

*Pregnancy-related aspects*

In hypertensive women planning to become pregnant, it may be prudent prior to conception to change to antihypertensive medications known to be safe during pregnancy, such as methyldopa or beta blockers. Pregnant women with stage 1 or 2 chronic hypertension without target organ damage should stop pre-pregnancy antihypertensive treatment while maintaining close observation, including use of home BP monitoring might be advised because of concern about the safety of antihypertensive drug treatment in pregnancy, including potential effect of BP-lowering treatment on fetal growth. Systolic/diastolic BP values of 150-160/100-110 mmHg mandate reinstitution of the previously withdrawn antihypertensive treatment in all pregnant women. An aggressive treatment of severe chronic hypertension in the first trimester is critical, since fetal loss rates of 50% and significant maternal mortality have been reported in these patients, Preferred agents to treat hypertensive disorders in pregnancy including the lactating period are methyldopa, or labetalol.

**Recent National Guidelines**

*2019 NICE guideline*

*Pregnancy-related aspects*

According to NICE [30], pregnant women should not use potassium chloride salt substitutes. Angiotensin converting enzyme inhibitors and angiotensin II receptor blockers should not be used in pregnant or breastfeeding women or planning a pregnancy women unless absolutely necessary, in which case the potential risks and benefits should be discussed. It is recommended that adults with type 2 diabetes of any age should start on an angiotensin converting enzyme inhibitor as step 1 treatment (except for women with a possibility of becoming pregnant and people of Black African or African–Caribbean family origin). For women considering pregnancy or who are pregnant or breastfeeding, management of hypertension should be in line with the recommendations on the management of pregnancy with chronic hypertension and antihypertensive treatment while breastfeeding in the NICE guideline on hypertension in pregnancy released in 2019 [41]. This guideline focuses on hypertensive disorders during pregnancy [41] and offers a clinical overview of hypertension disorders and lifestyle and drug interventions. Pregnant women at high risk of pre-eclampsia are advised to take 75–150 mg of aspirin daily from 12 weeks until the baby's birth. Women at high risk are those with any of the following: hypertensive disease during a previous pregnancy; chronic kidney disease; autoimmune disease such as systemic lupus erythematosus or antiphospholipid syndrome; type 1 or type 2 diabetes; chronic hypertension. Pregnant women with more than 1 moderate risk factor for preeclampsia are also advised to take 75–150 mg of aspirin daily from 12 weeks until the baby's birth. Factors indicating moderate risk are first pregnancy, age 40 years or older, pregnancy interval of more than 10 years, body mass index of 35 kg/m^2^ or more at the first visit, family history of pre-eclampsia, and multi-fetal pregnancy. A salt restriction is not recommended to prevent gestational hypertension or pre-eclampsia during pregnancy. Moreover, it is recommended that pharmacological treatment during pregnancy should stop if systolic/diastolic BP is lower than 110/70 mmHg or symptomatic hypotension emerges. Drug treatment should be offered to women with chronic or gestational hypertension and sustained systolic/diastolic BP >140/90 mmHg. The on-treatment BP target during pregnancy should be <135/85 mmHg. The guideline also suggest considering labetalol to treat chronic hypertension in pregnant women, nifedipine for women in whom labetalol is unsuitable, or methyldopa if both labetalol and nifedipine are both unsuitable. If treatment should be continued during the puerperium, methyldopa should be replaced by an alternative drug. Enalapril to treat hypertension in women during the postnatal period should be considered, with appropriate monitoring of maternal renal function and serum potassium.

*2016 Australian guideline*

*Sex and gender aspects*

The Australian guideline [31] acknowledge that individuals with hypertension often present additional risk factors, including male sex. The proportion of Australians with untreated or uncontrolled hypertension was greater in men than women (24.4% vs. 21.7%). It increased with age, peaking at 47% in individuals over 75. Family history of premature cardiovascular disease (immediate relative before 55 years of age for men and before 65 years for women) contributes to a higher overall risk than the estimated one. The recommendation for frequency and volume of alcohol was not different for men and women (no more than two standard drinks on any day and no more than four on any one occasion). *Pregnancy-related aspects*

Renin-angiotensin system blockers are contraindicated in pregnancy and should not be administered in women with childbearing potential. No recommendations were offered for pregnancy, but it was encouraged to refer to the 2014 Society of Obstetric Medicine of Australian and New Zealand (SOMANZ) guideline [40] addressing hypertensive disorders in pregnancy.

*2019 Japanese guideline*

*Sex and gender aspects*

The 2019 JSH report [36] provides different country-specific epidemiological aspects stratified by sex and sex-related recommendations. It is stated that the total mortality risk increased with the BP level exceeding 120/80 mmHg in men and women aged 40-89 years. An estimation on the basis of the results of previous epidemiological studies showed that hypertension is the most important factor of cardiovascular death in Japan and the average hypertension-related shortening of life expectancy was 2-3 years in men and women aged 40–49 years before adjustment for antihypertensive drug treatment during the follow-up period and thus, underestimated. The prevalence of hypertension is 60% for men aged 40–74, 41% for women in the same age range, 74% for men aged 75 and over, and 77% for women in the same age range. Based on the National Surveys, the prevalence of hypertension increased with age, exceeding 50% among men aged 50 and over and women aged 60 and over. The hypertension treatment rate has been rising during the past 36 years, exceeding 50% among men and women aged 60–69 and 60% among men and women aged 70–79. The hypertension control rate has been rising during the past 36 years but remains about 40% among men and 45% among women. The age-adjusted stroke mortality in Japan reached a peak in the 1960s and then fell sharply (1965: 361 for men and 244 for women, 2016: 36 for men and 20 for women [per 100000 population]). Regarding lifestyle attitudes in Japan, the salt intake evaluated by the weighing method in the National Health and Nutrition Survey has decreased gradually, and the mean daily salt intake per person was 9.9 g (men: 10.8 g, women: 9.2 g) in 2016. A sex-related recommendation was the reduction of alcohol intake: ≤20–30 mL ethanol per day in men and ≤10–20 mL ethanol per day in women. Regarding potassium intake by Japanese (age 20 and over), a unisex target of daily potassium intake (3000 mg or more) was proposed by Japanese health authorities. The Japanese report also highlighted another difference between men and women: the BP elevation due to moderate or more severe sleep apnea syndrome was evident in men but absent in women. The prevalence of metabolic syndrome among the Japanese aged 40 and over was 26% for men and 10% for women. There was no increase in the proportion of obesity in women aged 20 and over over the past 30 years (≈21% in 2016). However, from 1980 to 2010 contribution of obesity to hypertension rose from 11 to 27% in men and 19 to 26% in women. In Japan, the thresholds to define abdominal obesity are men ≥85 cm; women: ≥90 cm. There was a striking difference in hyperuricemia rate (urate level: >7 mg/dL or patients receiving urate-lowering drugs) between outpatient men and women with hypertension (40.6 and 8.6%, respectively). Although male sex is considered a risk factor for increased cardiovascular disease in the JSH guideline [36], the extent of reduction in the relative risk for cardiovascular diseases achieved by such antihypertensive treatment does not differ among sexes.

*Pregnancy-related aspects*

Hypertension in pregnancy is defined as BP >140/90 mmHg, while hypertensive disorders in pregnancy were classified into pre-eclampsia, gestational hypertension, superimposed preeclampsia, and chronic hypertension. Methyldopa and labetalol are recommended as first-choice drugs for the treatment of hypertension (chronic hypertension) at a gestational age of > 20 weeks. At 20 weeks and over, nifedipine may also be used at gestational age. If no other drug can be chosen and nifedipine is used at a gestational age of > 20 weeks, informed consent must be obtained from the patient before the drug use. For the treatment of gestational hypertension, hydralazine may be used as a first-choice drug in addition to the three drugs. It is recommended that long-acting nifedipine should be administered regardless of the dosage form only when the advantage of treatment exceeds its risk (for pregnant women after week 20 of pregnancy). The sublingual administration of capsule preparations should not be used. If eclampsia is present or suspected, magnesium sulfate should be intravenously administered. In pregnant women, angiotensin converting enzyme inhibitors, angiotensin II receptor blockers, and direct renin inhibitors should not be used.

*2018 Chinese guideline*

*Sex and gender aspects*

For the auscultatory BP measurements, the Korotkoff phase I and phase V are recommended to define systolic and diastolic BP, respectively [35]. However, in the case of children (< 12 years of age), pregnant women, patients with severe anemia, hyperthyroidism, aortic valve incompetence, or with the Korotkoff sound not disappearing, the Korotkoff phase IV (when the sound changes abruptly) as diastolic BP, is recommended. In the case of drinking, daily alcohol intake (better mild than strong wine) should not exceed 25 g for men and 15 g for women. Also, the weekly alcohol intake should not exceed 140 g for men and 80 g for women. Liquor, wine, and beer intake correspond to less than 50 mL, 100 mL, and 300 mL, respectively. For Chinese people, the guideline report defined excessive or harmful drinking for men and women (men, 41–60 g or >60 g or women 21–40 g or >40 g, respectively). In order to control body weight in a healthy range, waist circumference should be < 90 cm for men and < 85 cm for women. For people aged 35–64, the overweight rate is 38.8%, and the obesity rate is 20.2%, which is higher in women than men. According to the 2012–2015 National Survey [35], the prevalence of hypertension in young people aged 18 to 24, 25 to 34, and 35 to 44 years was 4.0%, 6.1%, and 15.0%, respectively, and it was higher in men than in women. The awareness, treatment, and control rate of hypertension were higher in men than women. For women with hypertension during pregnancy, the threshold for drug treatment initiation is BP ≥ 150/100 mmHg, while the treatment target is 150/100 mmHg.

*Pregnancy-related aspects*

However, drug treatment for hypertension in pregnancy can be withheld until BP ≥ 160/110 mmHg if the woman has no proteinuria or other target organ damage. In pregnant women with mild hypertension, non-pharmacologic intervention should be emphasized, with simultaneous monitoring of BP, proteinuria, or other target organ damage.

*2018 Korean guideline*

*Sex and gender aspects*

The 2018 Korean guideline for hypertension management [32-34] indicate that hypertension prevalence was higher in men (55.9%) than in women (46.2%) until the age of 60 but lower in men (64.2%) than in women (72.2%) after the age of 70. In outpatient Korean individuals, the prevalence of masked hypertension was 21.2%, and the male sex was an independent predictor of masked hypertension. The attributable risks to hypertension for cerebrovascular and coronary heart disease in Korean men were 35 and 21%, respectively. According to a Korean survey, the age-adjusted prevalence of hypertension first decreased from 30% in 1998 to 26% in 2009, then increased to 29% (men 35%, women 23%) in 2016. In addition, 25.9% of Korean individuals (men 31%, women 21%) have high-normal BP levels. Thus, 55% of Koreans aged 30 years or older have higher than normal BP. This percentage increases to 65.2% (men 61.7%, women 67.7%) among individuals aged 65 years or older, and the increase is greater in women than men. Using the criteria for abdominal obesity as a waist circumference greater than 90 cm for men and 80 cm for women, the prevalence of metabolic syndrome was 24.1% in a Korean survey. Among the components of the metabolic syndrome, high BP was the most common in men (40%), but low HDL-cholesterol level (59%) was the most common, followed by hypertension (30%) in women. The Korean guideline [32-34] support that hypertensive men ≥45 years old or women ≥55 years have a greater cardiovascular risk than their younger counterparts with no additional risk factors. However, a family history of premature cardiovascular disease is defined by different sex-related age thresholds (men <55 years, women <65 years). Abdominal obesity in Korea was defined as waist circumference >90 cm in men and >85 cm in women. As such, a waist circumference of less than 90 cm for men and 85 cm for women is recommended for Korean individuals. An appropriate moderate daily amount of alcohol is less than 20-30 g for men or 10-20 g for women. A man or woman with lower-than-average body weight is more sensitive to alcohol and is therefore permitted half of the recommended amount. There is no evidence for a differential hypertension treatment between women and men. Additionally, there is no difference in BP reduction and drug effects between women and men. Oral contraceptives can increase BP in some individuals, and the occurrence of accelerated or malignant HTN is rare. Therefore, BP needs to be carefully monitored in the early period of oral contraceptive use, while periodic measurements are recommended after that. After menopause, hormone replacement therapy is no longer recommended for cardiovascular prevention. Because such treatments can increase BP, women with a greater chance of developing hypertension should be carefully observed for a few months.

*Pregnancy-related aspects*

Hypertensive disorders in pregnancy include chronic hypertension, gestational hypertension, and preeclampsia superimposed on chronic hypertension. Based on BP levels, hypertension is classified as mild: 140-149/90-99mmHg, moderate: 150–159/100–109 mmHg, and severe: 160/110 mmHg or higher. In pregnancy, BP should be controlled below 150/100 mmHg, but it is not recommended to lower diastolic BP below 80 mmHg. Antihypertensive drugs used during pregnancy are methyldopa, labetalol, and nifedipine. Diuretics should be prescribed with caution because they can induce volume depletion. Renin-angiotensin system blockers may increase the risk of congenital malformations in pregnancy. It is recommended to replace these drugs before or when planning a pregnancy. If pregnancy is detected during the administration of renin-angiotensin system blockers, they should be discontinued and replaced promptly. Women with a history of preeclampsia have about a two-fold risk of ischemic heart disease, stroke, and venous thrombosis and a four-fold risk of developing sustained hypertension.
